# What Should Be Taught and What Is Taught: Integrating Gender into Medical and Health Professions Education for Medical and Nursing Students

**DOI:** 10.3390/ijerph17186555

**Published:** 2020-09-09

**Authors:** Hsing-Chen Yang

**Affiliations:** Graduate Institute of Gender Studies, Kaohsiung Medical University, Kaohsiung City 80708, Taiwan; yhc@kmu.edu.tw

**Keywords:** curriculum, gender concept, medical and healthcare professionals, sexism, structural competency

## Abstract

This study focused on gender education for medical and nursing students, because gender competency is essential for them to provide effective and appropriate healthcare and to promote equal rights to health. A questionnaire was administered to 50 health care professionals to explore the gender concepts and gender knowledge that they deem imperative and often teach to medical and nursing undergraduate students in class. Sexism, gender awareness, sexual harassment, the topics of three acts related to gender equity, and patriarchy are the gender concepts participants deemed most crucial for students to learn and understand. However, disparities were noted between the gender concepts frequently taught by the participants and the gender knowledge they considered essential for students. The 50 experts emphasized teaching the concept of patriarchy and the cultivation of students’ structural competency in addition to identifying directions for gender, medical, and health care education. By highlighting the key gender-related concepts, the present research findings may benefit teachers who intend to integrate gender into the curriculum but are limited by time constraints. The results offer a professional development direction for teachers endeavoring to incorporate gender into the curriculum and their teaching.

## 1. Introduction

Awareness and knowledge of gender has been acknowledged to constitute a key competency among medical and healthcare professionals. The value of integrating gender into professional health education is therefore recognized [1,2,3]. However, gender is currently considered ‘important…but of low status,’ occupying a marginal position in medical education [1]. A disparity between ideals and reality, caused by equating gender with biological sex and the treatment of gender neutrality as a naturally occurring phenomenon, is associated with inadequate understanding of gender. This creates challenges in relation to the inclusion of gender in medical and healthcare education [1,4,5].

Medicine and healthcare are specialist areas, and so is gender education. In addition to the proper delivery of professional knowledge, communications between doctors and nurses and patients often require understanding of and sensitivity to gender, necessitating some gender knowledge and gender competency. Studies have shown that lesbian, gay, bisexual, transgender, queer, and intersex (LGBTQI) people, having lived in a heterosexual society for a long time, tend to accumulate mental stress. When they face medical staff, they often feel awkward and difficult to reveal their true self [6]. Some LGBTQI people are highly private regarding their bodies. When seeking medical treatment, they may experience feelings of discomfort and fear or show resistance when asked to expose or allow medical staff to touch their bodies during physical examination [6,7,8]. Insufficient gender sensitivity or knowledge of LGBTQI culture among medical staff can inflict secondary injuries on the patient and impede disease diagnosis and treatment.

This study asserts that the right to equal health is a matter of concern in medicine and healthcare, as well as a topic to be addressed through education because changes in culture and in values are required rather than mere systematic and policy changes. Education is always crucial in the training of healthcare professionals. This is why multiple scholars have advocated the incorporation of gender into medical and healthcare education [2,4,9,10,11].

Undergraduate education is the preliminary stage in the career paths of various professionals. To promote equal health rights, crucial professional competencies for medical and nursing students should include, but not be limited to, gender competency. Eliminating health disparity and practicing gender-friendly medical care requires undergraduate gender education for medical and nursing students. For medical and nursing students, exploration and construction of the core gender-related concepts facilitate the development of gender competency.

This study surveyed medical and nursing teachers and healthcare professionals to determine what gender knowledge they deemed crucial for medical and nursing undergraduate students and which gender-related concepts they most frequently taught. This study provided answers to the research question “Which gender-related concepts should be incorporated into curricula to prepare students for the future?”

### 1.1. Gender Education in Medicine and Healthcare: Reform of the Curriculum and Knowledge Enhancement

Curriculum reform is the core of education reform. Multiple studies have explored the effectiveness of incorporating sex and gender into medical and healthcare courses [4,5,11,12,13]. Medical education in the Netherlands may be considered as an example. Under the leadership of the research team at Radboud University Nijmegen Medical Center, eight medical schools in the Netherlands implemented the gender curriculum plan developed by the research team to promote gender education. Moreover, a set of indicators was developed to assess the inclusion of gender issues in medical education courses. Examples of the indicators are as follows: inclusion of sex and gender differences in medical education objectives; discussion of gender in the context of professional medical settings with biomedicine and sociocultural structures also taken into account; and consideration of factors relating to gender, age, social class, and race in discussions of human health and disease [14].

Experiences of integrating gender into medical and healthcare education in Australia, Sweden, the United States, Canada, Taiwan, and other countries and territories also demonstrates the benefits of gender courses and the manner in which they contribute to students’ understanding. For example, after studying gender, students can: recognize gender differences; address medical problems from a gender perspective; demonstrate awareness of the influence of social and cultural factors on individual health, which is generally believed to be affected only by biomedicine; and demonstrate increased sensitivity to the healthcare needs of LGBTQI patients [9,10,15,16,17,18].

The following measures were adopted during medical education reform in Taiwan: In 2002, the Ministry of Education published the White Paper on Medical Education in which “enhancing gender equity in medical education” was declared to be an objective. In 2007, the Ministry of Health and Welfare revised the Regulations Governing Practice Registration and Continuing Education of Doctors to incorporate gender issues into the curriculum. In 2013, the Taiwan Medical Accreditation Council added gender to the educational evaluation criteria of the new accreditation standards and demanded cooperation between teaching hospitals and universities to provide students with education and training regarding gender equity regulations and to ensure their understanding of key gender issues [19].

Development of the aforementioned national education policies has resulted in administrative measures and regulations in relation to the incorporation of gender into the education system and curricula. However, key topics in relation to gender require identification. Studies have proposed that the integration of gender into curricula and professional health education is challenging for the following reasons: problems achieving conceptual clarity; lack of time and space to accommodate gender in the curriculum; skepticism regarding the incorporation of gender into the curriculum; lack of time or willingness to learn about gender among teachers; gender blindness in the medical system and knowledge; treatment of women’s issues as gender issues and negligence of the existence of multiple genders; the categorization of gender in childbirth topics or obstetrics, strengthening of the link between women’s health and reproduction [1,3,4,5,14,20,21,22].

Studies have also shown that the lack of concrete gender-related curricula, gender-related theories, or comprehensively addressed gender-related topics has rendered gender education in medicine difficult [1,3]. Risberg et al. [1] noted that inadequate knowledge of gender is a major obstacle to the integration of gender into curricula among male teachers who also serve as leaders of medical universities. Risberg et al. discovered that these male teachers: (1) lacked perception of subtle inequalities between men and women; (2) did not know which areas to address in gender education; and (3) believed that gender education was merely the discussion of the physiological and behavioral differences between men and women, but lacked the time to engage in gender studies. An improvement strategy proposed by Risberg et al. was that gender be considered an individual area of scientific knowledge. To achieve this, the Medical Education Committee or course directors require sufficient understanding of the aspects of gender that should be included in courses to clearly define learning goals and properly allocate time for studying each aspect of gender.

The aforementioned research revealed that, although policies and curricula are being reformed to incorporate gender into professional health education, additional efforts are required for further improvement. To achieve further improvement, the curriculum must be reorganized and the structure of gender-related knowledge reformed to identify the gender-related concepts and concerns that should be taught in health professions education as well as enhancing teachers’ professional knowledge of gender.

### 1.2. Feminist Lens, Gender Theory, and Gender Learning

The women’s health movement emerged in the 1990s, originating from the awareness of gender bias in a male-dominated medical enterprise [23]. In such settings, women’s health issues, life experiences, and social situations are excluded from the construction of medical knowledge, as well as being neglected in clinical medicine and healthcare.

The central tenet of both feminism and gender theory is how to end sexism, sexist exploitation, and oppression [24,25]. Feminists criticize the male-centered values in biomedicine, which they associate with patriarchy [23,26]. According to sociologist Allen Johnson [27], patriarchy implies male-centeredness, male dominance, and male identification. Sharma [23] noted that patriarchy has ripple effects such as gender segregation in medical specialties and leadership, a gender pay gap, and harassment.

Influenced by feminism, gender theories, and the women’s movement, Taiwan passed the Gender Equity Education Act in 2004, implementing gender equity education in schools at all levels through institutionalization. Stromquist [28] examined the gender education policies implemented by countries worldwide and commented on the distinctive and progressive nature of Taiwan’s Gender Equity Education Act on a global scale. The act focuses on the cultivation of gender awareness through gender education as well as through the elimination of sexism and sexual harassment on campus. Taiwan’s three acts on gender equity, namely the Gender Equity Education Act, the Gender Equality in Employment Act, and the Sexual Harassment Prevention Act, all share a common emphasis on the elimination of sexism and sexual harassment. In particular, the Gender Equality in Employment Act guarantees equal work rights for all genders, prohibits sex discrimination, prevents sexual harassment in the workplace, and focuses on measures to promote work equality and the establishment of a gender-friendly environment.

Knowledge of Gender Equality in Employment Act and of gender-related regulations is mandatory in the education and training of medical and nursing students during internships governed by the Taiwan Medical Accreditation Council and in the gender curriculum for the continuing education of doctors and nurses, per Ministry of Health and Welfare guidelines. Both education and training programs focus on developing medical and nursing students’ and medical personnel’s awareness of power structure relationships and of the right to work and medical rights. Sharma [23] noted that the application of feminism in medical education and curricula can help to eliminate gender stereotypes and sexism in medicine and clinical practice as well as ultimately resolving various forms of gender inequality, such as sexual harassment. For example, understanding power and privilege improves teachers and students’ awareness of power structures and power relations in medicine and clinical practice, as well as the health disparity among men, women, and LGBTQI people. Moreover, the application of feminism or gender theories to professional health education and students’ study of gender contributes to the creation of more systematic training in professional health education, as advocated by many studies. Healthcare professionals can thereby determine how the interplay of gender, race, social class, and sexual orientation affects individual health, while recognizing how social structures and economic and inequality factors operate in a larger structural environment to provide patients with superior care and humane medical practices [29,30].

## 2. Methods

In this study, a questionnaire survey enrolled 50 medical or nursing teachers and healthcare professionals. Taiwan’s policies, systems, and organizations in relation to gender equality education have contributed to gender education, as reflected in the reform of university curriculum structures, professional certification, and professional continuing education. Many medical universities or medical/nursing programs have established gender-related courses or have integrated gender into their curricula. The government also requires that courses on gender be included in continuing education for medical personnel.

### 2.1. Survey Questionnaire

The design of this questionnaire was based on research purposes and questions. The questionnaire items queried respondents on the gender-related concepts that they frequently teach and consider essential for students’ learning and knowledge. Respondents were asked to consider medical/healthcare specialties and occupational competency in completing the self-administered questionnaire. The research team mailed or emailed the questionnaires to the participants for them to complete independently.

The questionnaire was prepared, and expert validity was used to test the content validity of each item. Three gender education experts and scholars tested the validity of, reviewed, and revised the questionnaire. The revised questionnaire included two parts: (1) demographic data; and (2) questions on gender education for students. The second part of the questionnaire was composed of open-ended questions, multiple choice questions, and sequential questions. The sequential questions involved prioritizing and ranking multiple items by their perceived importance. The second part’s questions were as follows:

1. Please list at least three gender concepts or gender knowledge that you often teach in your course or at work.
2. Among the 25 items (the 25th item is “other”), please select 15 gender concepts or gender knowledge that university students must learn (if you select “other,” please specify its content).
3. Continuing from Question 2, please rank the 15 gender concepts or knowledge according to the following three major categories: most important and highest priority, second most important and second highest priority, and least important and lowest priority; please also explain your reasons for the ranking.
4. Taking the department you teach in or work competency into consideration, among the 25 items, what do you think are the 15 gender concepts or gender knowledge that students must learn to succeed in future workplaces and in their social life?
5. Continuing from Question 4, please rank the 15 gender concepts or knowledge according to the following three major categories: most important and highest priority, second most important and second highest priority, and least important and lowest priority; please also explain your reasons for the ranking.
6. Please provide any further opinions or suggestions on the gender concepts or related knowledge that students must learn and know.

### 2.2. Participants, Data Collection, and Data Analysis

This study employed purposive sampling and ensured the representativeness of expert opinions by establishing a sampling standard, which addressed the professional knowledge or experience of the participants in the professional fields of gender, medicine, and healthcare. The inclusion criteria required that participants demonstrate gender awareness and possess years of teaching or practical experience in medicine and healthcare, in addition to meeting any of the following requirements regarding professional knowledge: (1) they were teaching courses on gender and medicine or healthcare at universities or had incorporated gender into medicine or healthcare courses; (2) they had published works related to gender and medicine or healthcare; (3) they had conducted research projects on topics related to gender and medicine or healthcare; or (4) they had promoted gender education for medical personnel or gender-friendly medical care.

Participants were recruited through the following channels: (1) the researcher’s interpersonal network; (2) professional associations or gender-related nongovernmental organizations; (3) a search for university professors teaching courses related to gender and healthcare; (4) a search for researchers implementing gender- and healthcare-related research projects commissioned by national institutes; and (5) a search of the various agendas of gender-related conferences/seminars to establish a list of authors who had published papers on gender, medicine, and healthcare. This study used the snowball sampling technique to send invitation emails to those satisfying the sampling criteria.

This study recruited 50 participants, 35 of whom were teachers and 15 of whom were healthcare professionals. Regarding the sex of participants, 18 were males (including one female-to-male transsexual person). Of the participants, 4, 14, 13, 15, and 4 people were aged 20–30 years, 31–40 years, 41–50 years, 51–60 years, and 61 years or older, respectively. Notably, many of the participants teaching in the department of medicine were also licensed doctors. Twenty eight of the participants specialized in medicine, whereas the remainder specialized in nursing.

For data coding, the following methods were adopted separately according to the nature of the open-ended questions: (1) Record participants’ answers. For example, for the sixth question in Section 2.1, this study accurately recorded the answers provided by participants. (2) Categorize and encode participants’ answers. The “other” item in the multiple-choice questions may be used as an example. This study originally provided 25 concept items, one of which was “other.” The “other” answers provided by the participants were categorized according to frequency. A new gender concept code was established when a concept was mentioned by different participants and more than three persons; a total of four new gender concepts were established. These gender concepts were gender role stereotypes, gender traits, feminism, and gender mainstreaming. Non-repeated concepts were still categorized as “other.” A total of 30 gender concepts emerged after data encoding.

For data analysis, this study used different methods for qualitative and quantitative data. Clustering, counting strategy, and thematic analysis were used for qualitative data; descriptive statistics and correspondence analysis were used for quantitative data. For sequential questions, the study adopted a weighted scoring method to assign 15, 10, and 5 points for items deemed most important (top priority), less important (secondary priority), and least important (third priority), respectively. The importance and priority scores for the multiple choice questions were compared. For correspondence analysis, 30 items were combined and divided into four groups through an expert validity test: (1) patriarchy and heterosexuality: heterosexual hegemony, heteronormativity, patriarchy, misogyny, homophobia, male-centeredness, male-dominance, male-identification, objectification, patriarchal dividend; (2) sex and gender: LGBTQI, gender awareness, sexual and gender identity, gender traits, sexism, gender role stereotypes; (3) gender violence: sexual harassment, sexual and gender-based bullying, domestic violence, oppression and gender oppression, abuse of power; and (4) gender politics: exploitation, Taiwan’s three acts on gender equity, autonomy, emotional labor, body politics, feminism, gender mainstreaming, and intersectionality.

### 2.3. Ethical Considerations and Approval

This research complies with the basic principles of research ethics, including informed consent, privacy, autonomy, and protection from harm. The invitation letter clearly explained the research purpose, research questions, questionnaire survey method, and data processing method, including the anonymity of the survey, the number of questionnaire items, the estimated completion time, and the steps required for the termination of participation. Only after consent was obtained from potential participants would the questionnaire and informed consent form be sent out. Therefore, the invitees fully understood the use of and processing methods for research data (e.g., the anonymous processing of data) before deciding, with full autonomy, whether to participate in the study. The National Cheng Kung University Human Research Ethics Committee approved the anonymous questionnaires.

## 3. Results

### 3.1. “Sexism” Ranks First among the Gender Concepts That Medical and Nursing Students Must Learn and Understand

Based on medical and healthcare specialties and occupational competency, the 50 experts identified the top 15 gender concepts essential to medical and nursing students’ learning and knowledge, with the top 10 being sexism, sexual harassment, gender awareness, patriarchy, the three gender equity acts, LGBTQI, sexual and gender-based bullying, sexual identity and gender identity, autonomy, objectification, and oppression/gender oppression (Table 1). Up to ≥80% of the participants deemed that medical and nursing students must learn and understand “sexism” and “sexual harassment.”

The top three gender concepts considered essential for medical and nursing students to learn and understand were the same for male and female participants: sexism, sexual harassment, and gender awareness. The top 10 gender concepts proposed by male and female participants were relatively similar, with the only difference being that female participants included objectification and body politics, whereas male participants did not. The top three gender concepts proposed by teachers and healthcare professionals varied; the concepts highlighted were sexism, gender awareness, and sexual harassment and patriarchy, sexism, and sexual harassment, respectively. Among the top 10 gender concepts proposed by teachers and healthcare professionals, objectification and body politics were ranked as crucial only by teachers, whereas “oppression and gender oppression” was ranked as crucial only by healthcare professionals.

Notably, up to 93% of healthcare professionals believed that students must learn and understand the concept of “patriarchy.” Eighty percent of the participants working as teachers or healthcare professionals identified the gender concept of “sexism” and “sexual harassment” as essential for medical and nursing students to learn and understand.

**Figure 1:**
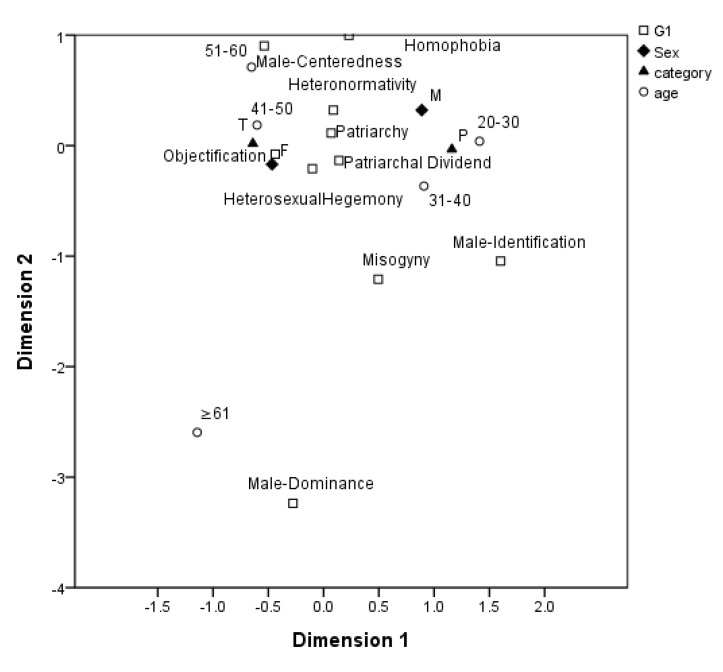
Distribution of gender concepts within the “patriarchy and heterosexuality” grouping.

Correspondence analysis was used for this study to explore the relationship between demographic variables and concept combinations; the variable of age was added to identify whether generational differences existed. Figure 1 shows the analysis results for the “patriarchy and heterosexuality” grouping. The selections of patriarchy, patriarchal dividend, and heterosexual hegemony were not subject to variable changes. That is, all participants preferred these gender concepts essential for students to learn and understand. Male and female participants preferred that students learn and understand homophobia and objectification, respectively. Teachers and healthcare professionals had no particular preferences for certain concepts. Participants aged 20–40 years, 41–50 years, 51–60 years, and older than 61 years preferred that students learn and understand “male identification and misogyny,” “objectification,” “male-centeredness,” and “male dominance,” respectively.

**Figure 2:**
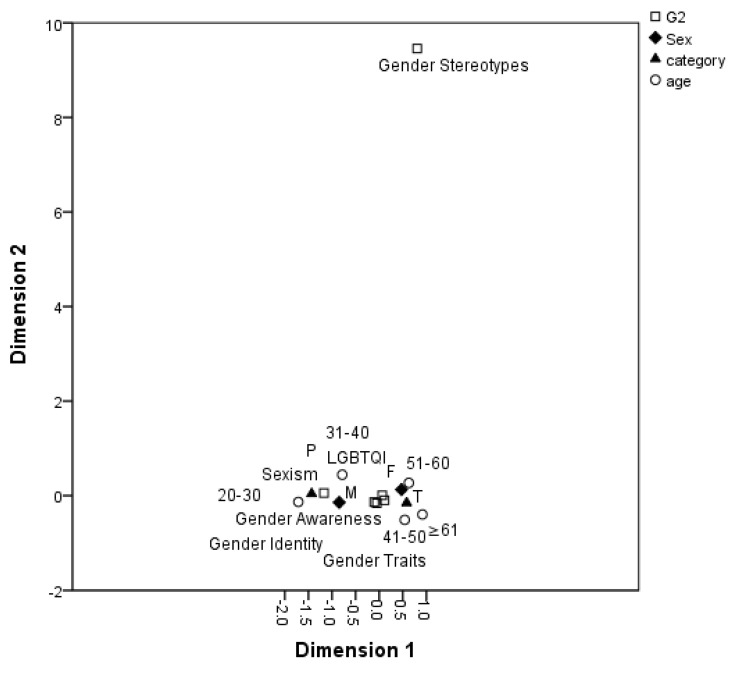
Distribution of gender-related concepts within the “sex and gender” grouping.

Figure 2 depicts the analysis results for the combinations of concepts within the grouping for “sex and gender.” Overall, most participants preferred students to learn the gender-related concepts within this group. In the correspondence analysis plot, most concepts are in close proximity with one another, and no considerable differences exist. In particular, the participant selections of sexism, sexual identity and gender identity, gender awareness, gender traits, and LGBTQI were not subject to variable changes. That is, participants regarded these gender concepts essential for students to learn and understand.

**Figure 3:**
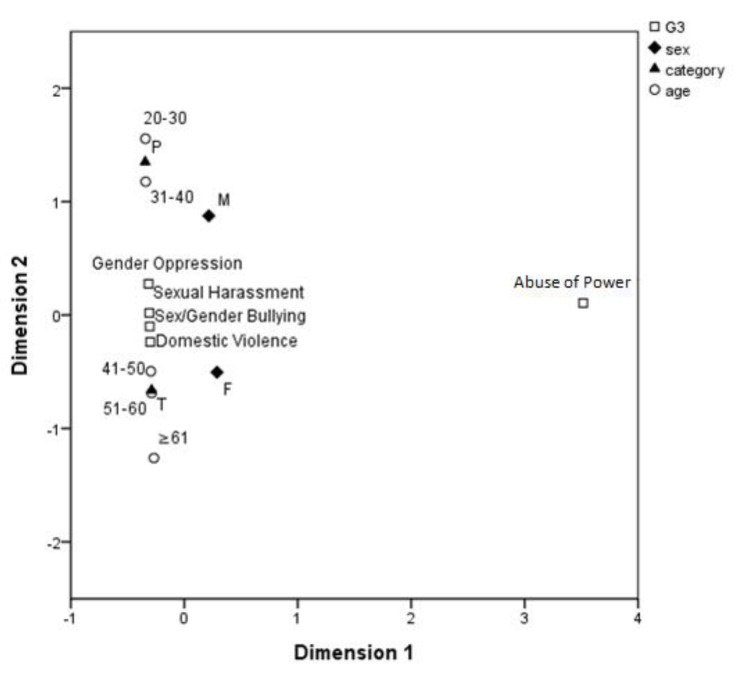
Distribution of gender concepts within the “gender violence” grouping.

Figure 3 depicts the analysis results for the combinations of concepts within the grouping for “gender violence.” Sexual harassment, domestic violence, sexual and gender-based bullying were not subject to variable changes. That is, participants regarded these gender concepts essential for students to learn and understand. Male participants, healthcare professionals, and participants aged 31–40 years all preferred that students learn oppression and gender oppression. No notable differences were found among the remaining data.

**Figure 4:**
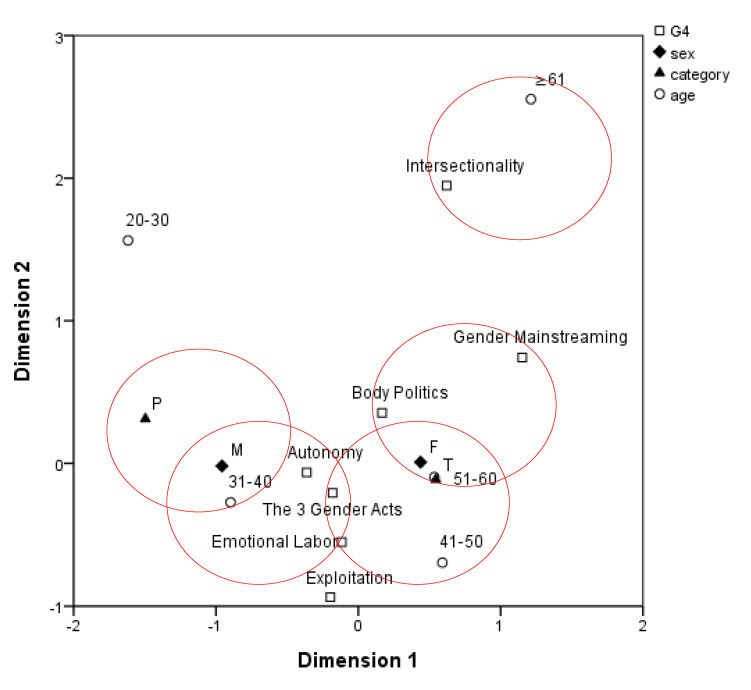
Distribution of gender-related concepts within the “gender politics” grouping.

Regarding “gender politics” (Figure 4), the majority of participants regarded “autonomy” as an essential gender-related concept for students to learn and understand. In the correspondence analysis plot, male participants are in close proximity to exploitation, the three acts on gender equity, and emotional labor; female participants are in close proximity to body politics and gender mainstreaming. Teachers are in close proximity to body politics and gender mainstreaming; healthcare professionals are in close proximity to the three acts on gender equity. Participants aged 31–40 years are in close proximity to gender mainstreaming and emotional labor; participants older than 61 years are in close proximity to intersectionality. The remaining data showed similar distribution patterns.

### 3.2. Gender Concepts Prioritized for Students’ Learning and Knowledge

Table 2 shows the ranking of gender-related concepts in terms of their importance for students’ learning and knowledge. Based on medical and healthcare specialties, occupational competency, and importance, the participants rated gender-related concepts on a scale of 1–3. The majority of the participants rated sexism, gender awareness, sexual harassment, the three gender equity acts, and patriarchy as the most important gender concepts for medical and nursing students. LGBTQI, sexual and gender-based bullying, sexual identity and gender identity, and autonomy were deemed less important. Finally, the gender concepts ranked least important were: objectification, domestic violence, oppression/gender oppression, heterosexual hegemony, homophobia, and body politics.

Male and female participants generally had the same opinions concerning the top-priority gender-related concepts; participants of both genders agreed that teaching sexism, gender awareness, sexual harassment, and three gender equity acts were priorities, but male and female participants respectively mentioned LGBTQI and patriarchy as top-priority gender-related concepts. Teachers and healthcare professionals both identified the following gender concepts as crucial for medical and nursing students: gender awareness, sexism, sexual harassment, three gender equity acts, and patriarchy. That is, these concepts were deemed gender education priorities. Notably, concepts classified as “other” were considered less important (of secondary priority) according to participants’ responses, regardless of gender and occupation. This phenomenon is subsequently explained further with reference to the qualitative data for participant responses.

### 3.3. Reasons for Prioritizing Gender Concepts According to Levels of Importance

The majority of the participants stated that basic gender knowledge required for medical professionals is the most important gender concept that should be prioritized in students’ education. Advanced learning is implemented after students master basic gender-related concepts and knowledge. They are of secondary importance or even lower priority, and are only taught when the class schedule or time permits. Specifically, some gender concepts are ranked low priority by some teachers because these concepts are more advanced and abstract, such as the concepts of heteronormativity and body politics.

Thematic analysis results showed that participants listed certain gender concepts as the highest priorities in gender and medicine education for students for the following three reasons:

(1) Learner orientation (centering on students’ personal and workplace practices): Participants, whether in the field of medicine or nursing, believed that the purpose of their professions and jobs were to serve people. Therefore, an understanding of certain basic gender-related concepts, such as sexism, gender awareness, and sexual harassment is equally crucial to students and to their future care receivers. Participants responded that emphasizing gender knowledge enhances personal and medical profession–related gender competency in students as well as facilitates the establishment of a gender-inclusive medical environment. For example, Ada, a female teacher in the department of nursing stated, “I teach a course entitled “Gender and Nursing.” My students are about to conduct internship in hospitals, so I teach them manifestations of sexual harassment prevalent in the nursing workplace, thereby preparing them for their internships at hospitals.” Another female teacher, Betty, in the department of nursing also responded, “Nursing students should be more sensitive to and possess more understanding of gender issues in the clinical care of patients, including potential sexual harassment incidents and sexism in spoken language.” Cathy, a female teacher teaching in a medical school remarked, “I teach in a medical school, and a notable gender gap exists both in school and in the future workplace. I hope to clarify the causes of such a phenomenon for students.”

(2) The value of understanding structural inequality: Participants emphasized that medical and nursing students must understand the inequality in social structures and gender relations and their effects on gender culture and gender interaction. This also explains why the majority of participants ranked “patriarchy” as the most crucial gender concept to be prioritized in students’ education on gender. A female teacher, Daisy, in the nursing department stated, “The top priority is for students to perceive the existence of gender inequality in social structures and to recognize the comprehensive influence of patriarchy on culture and society. Particular attention should be devoted to inequality between men and women, which renders women relatively vulnerable. Such awareness is the first step for understanding gender-related concepts or for acquiring gender education.” The only transgender participant, Terry, in this study responded “Patriarchy deeply influences all aspects of daily life. I believe that it is the main cause for gender inequality and the existence of oppressive and dominant relationships in society. Therefore, I will prioritize [the learning] of it [patriarchy].”

Furthermore, gender inequality, power structures, and power relations in clinical practice, as well as their potential influence on students in their future workplaces, are the main reasons for which participants listed “patriarchy” as the most crucial gender concept in gender education. A male doctor, John, remarked on the value of understanding patriarchy, saying that “This gives undergraduate students an overall picture of their future workplaces, enabling them to quickly grasp gender dynamics within it.” A female doctor, Ella, mentioned, “The surgical department is a patriarchal working environment… It’s male-centered….homosexuality is…highly frowned upon in such a work environment.”

(3) By prioritizing certain gender concepts in undergraduate education, participants hope to improve students’ understanding of gender-related regulations to protect their rights and their patients’. This objective is also related to the aforementioned two reasons. A female teacher, Erica, in the long-term care profession said, “Acts guarantee fundamental rights, so the three acts on gender equity are must-knows… Students must also learn about sexual harassment, which is common in the care industry.” A nursing department teacher, Fanny, said, “We can start fostering gender equality on campus or in the workplace by teaching the three acts on gender equity. Specifically, the Gender Equality in Employment Act targets the prevention of sexual harassment in the workplace, and the Gender Equity Education Act focuses on the prevention of sexual harassment incidents on campus.” Paying particular attention to the effect of Taiwan’s same-sex marriage legalization on LGBTQI medical rights and medical decision-making, another nursing department teacher, Gail, indicated that students must understand policies and measures related to LGBTQI medical rights.

Overall, the interrelated connection among the aforementioned three reasons indicates that the participants planned a bottom-up learning approach to gender education by imparting the fundamental concepts first. This study also reveals a notable phenomenon: Although the participants were asked only to provide reasons for how they prioritized and ranked gender-related concepts, most participants also explained how they should or would teach crucial gender concepts that should be prioritized in education. For example, a male doctor highlighted the importance of learning the three gender equity acts and stated, “I think that studying gender-related regulations is necessary. Interactive case-based discussion, debate, and dialectic can be employed to avoid the dullness of learning regulations. After all, the real purpose of education is to address the values behind each article, rather than mere memorization and lecturing.”

### 3.4. Frequently Taught Gender Concepts or Knowledge

Most participants reported that the gender concepts they frequently imparted to students were basic gender concepts or knowledge requiring prioritization in learning. However, considerable disparities and contradictions were noted in this study between the aforementioned statistical results and the qualitative responses provided by participants.

Because participants provided various types of answers to the question regarding gender concepts they imparted most frequently, a counting method was used to organize these. A total of 58 gender concepts were identified, none of which was simultaneously mentioned by ≥50% of the participants. Only the following four gender concepts were simultaneously mentioned by ≥20% of the participants: gender diversity, patriarchy, gender role stereotypes, and sexual harassment (in descending order).

The analysis results demonstrated that the gender concepts and knowledge frequently taught by participants did not converge on specific concepts. Although most participants provided more than three gender-related concepts, their answers—which were widely dispersed—rarely overlapped or intersected. Gender diversity, the most frequently mentioned gender-related concept, was mentioned by only 24% of the participants. Concepts of patriarchy, gender role stereotypes and sexual harassment, LGBTQI, and sexism were frequently taught by 22%, 20%, 16%, and 14% of the total participants, respectively.

Gender knowledge comprises either “one or several gender concepts” or “a specific conceptual category or theme relating to gender,” such as gender medicine. In this study, only two female teachers reported “gender medicine” to be a gender-related concept that they often imparted to students. Both taught in the department of medical humanities and specialized in feminism, gender sociology, and gender research. To enhance medical students’ competency in social sciences and humanities, medical universities in Taiwan recruit teachers with medical humanities backgrounds and establish courses on medical ethics and social sciences. The only female teacher who mentioned “gender oppression” and “power relations” as concepts she frequently teaches served as a medical humanities teacher at a medical university and was one of the few teachers integrating feminism into the curriculum.

## 4. Discussion

### 4.1. Consensus: Sexism, Gender Awareness, and Sexual Harassment Are Core Gender Knowledge Required of Students

The 50 medical and nursing professionals appeared to have a clear idea of the gender knowledge that medical and nursing students should learn. They also achieved a high level of consensus on gender concepts that should be prioritized in gender education for students: sexism, gender awareness, and sexual harassment. Simply put, participants identified sexism, gender awareness, and sexual harassment as core gender knowledge for medical and nursing students, and as essential for students to learn and understand.

The gender concepts of patriarchy, patriarchal dividend, and heterosexual hegemony were also mutually identified by respondents as essential for students’ learning and knowledge. The male-centeredness or male-dominated organizational structure and culture of the medical workplace were the reasons for which participants selected patriarchy or sexism, gender awareness, and sexual harassment as crucial gender knowledge requiring prioritization. A female teacher in the medical department said, “Medical students must understand the power relations between doctors and patients, recognize the patriarchal structure of society, respect autonomy, and avoid sexism in the process of caring for patients.” The three concepts in question were also emphasized in the conclusion of Metzl and Hanse [30], who indicated that medical and healthcare education should cultivate structural competency by switching focus from inequality in health between individuals to structural factors causing health disparity.

Qualitative data from the survey revealed that participants were mutually concerned regarding the development of gender awareness in students to prevent them from becoming victims or perpetrators of sexism or sexual harassment. In particular, female participants showed more concern in their qualitative data regarding gender discrimination and sexual harassment that medical and nursing students might have encountered in their future workplaces as well as regarding the corresponding gender education and learning. The correspondence analysis result showed that female participants preferred students to study the following gender concepts and knowledge: objectification, body politics, and gender mainstreaming.

In response to the question “Is gender or gender education a woman issue?” this study asserts that gender matters in medical school and in clinical practice. In Taiwan’s medical field, female nurses have a minority status despite being the majority, whereas female doctors are the minority in both number and status. To exacerbate matters, severe gender division exists in both the medical and nursing departments. Numerous studies have addressed the influence of gender bias and sexism on the learning, work, and career development of female medical students. Female medical students and teachers continue to experience sexism in medical school and clinical practice [3,31]. Male and female nursing students also experience similar situations and encounters on campus and in the workplace [32,33,34,35].

Gender bias and sexism in medical schools and the workplace require attention and redress. The elimination of sexism, the improvement of gender awareness, and the reformation of patriarchy are the core tenets of feminism and gender theory [23,25,36]. Lagro-Janssen [37] noted that core gender knowledge must first be established to achieve gender equality in the medical system. Accordingly, the identification of gender concepts involved in common gender-related problems and concerns in clinical practice and the incorporation of these concepts into core gender knowledge (established based on gender-related theories) can more effectively promote the integration of gender into medical and nursing curricula and promote effective teaching and learning.

### 4.2. Contradiction: Disparities between the Content Most Frequently Taught and That Considered Most Valuable in Students’ Learning

The aforementioned results revealed considerable disparities between the gender concepts most frequently taught and the gender knowledge deemed crucial to students. Specifically, sexism, gender awareness, sexual harassment, the three gender equity acts, and patriarchy were identified by participants as essential gender knowledge for medical and nursing students. However, only approximately 20% of participants frequently taught concepts of “patriarchy” and “sexual harassment.” Although participants regarded “sexism” as a top-priority gender concept, they rarely taught it in class. The awareness, one of the gender concepts prioritized by participants, was not even included among the gender concepts frequently taught by participants. Only one nursing teacher reported teaching the three acts on gender equity frequently.

A significant difference was also observed in the numbers of teachers and of healthcare professionals who selected the concept “other.” Teachers also ranked “other” as a gender concept of less importance (secondary priority) for medical and nursing students. “Other” is a general term for nonrepetitive gender concepts; that is, “other” encompasses various gender-related concepts. This may explain the diverse responses of participants in response to the question regarding which gender concepts they taught most frequently.

Integrating gender issues into the curriculum is a common method for medical and health professions education [10,11,14]. The advantage of the gender-integrated curriculum lies in the incorporation of crucial topics related to gender, to which students may rarely be exposed, into courses for existing subjects [38]. The integration of gender into medical or nursing curricula, for example, allows for the combination of gender equality education–related ideas, goals, and concepts with the objectives of medical and nursing education. This enables teachers to implement gender education into medical or nursing courses. Accordingly, a significant task for teachers designing gender-integrated curricula is the selection of core gender-related concepts and knowledge and their integration into the course objectives for other subjects. This prevents the tension generated during the integration of interdisciplinary subjects or the marginalization of any field of study. Teachers’ level of knowledge in the field of gender is the key to successful incorporation of gender into teaching. Teachers who lack sufficient understanding of gender education cannot change the minority status (i.e., ignored in the hierarchy of knowledge and values) of gender in the medical knowledge system [39]; they also slow down students’ learning in relation to gender. Therefore, curriculum designers or educators require an understanding of the core gender-related concepts and knowledge required of medical students to successfully promote the development and teaching of gender-integrated courses.

In addition, both policy and social changes affect knowledge construction. Medical and healthcare education, which is subject to social trends and contexts, is continually changing. The challenges in relation to the emergence of gender as an area of study also require innovative knowledge and discourse. Gender medicine is an interdisciplinary and relatively new subject that has gained much attention from medical and health educators [3,26]. However, this study revealed that only two teachers have introduced “gender medicine” in their classes. Taiwan has yet to treat gender medicine as an interdisciplinary professional field of study or to include it in the study of gender.

### 4.3. Divergence: Why Do Gender Stereotypes Continue to Exist in Teaching?

Surprisingly, gender stereotypes were ranked as the second most frequently taught gender concept by participants. Undergraduate medical and nursing students in Taiwan grew up after the implementation of the Gender Equity Education Act, which means that they began receiving gender equity education—with gender stereotypes being the first concept they studied—at elementary school. The question meriting discussion is whether imparting such fundamental gender concepts to these undergraduate students stimulates learning motivation and produces favorable learning results.

A nursing department teacher, Iris, stated, “Nursing students often face the problem of gender stereotypes in their subsequent professional lives providing clinical care.” A female doctor, Hazel, explained her reason for selecting “gender stereotypes” as a top-priority gender concept priority: “Gender stereotyping and discrimination occur frequently, and relevant education serves as a tool for understanding such phenomena.” Meulen et al. [2] compared the gender curricula developed by the Nijmegen Medical Center in 2005 and 2014 and reported that “gender stereotyping directed at patients” was a gender issue retained in the curriculum. Multiple studies have revealed that gender stereotypes continue to exist in medical school and clinical practice, and that medical or healthcare students and healthcare professionals have experienced gender inequality [3,31,40].

Further investigation is required to determine why gender stereotypes are still perpetuated by one-third of teachers or medical professionals. This study uncovered useful clues and meaningful teaching guidance from participants’ responses. If medical and nursing teachers or practitioners still intend to teach gender stereotypes in their curriculum, they may include the concept of unconscious bias to enrich and deepen the teaching and discussion of the concept. In addition, as already stated, participants considered the gender-related problems and concerns of students in school education and in their future workplaces to be crucial and thus emphasized the understanding of sexism, sexual harassment, and gender awareness among medical and nursing students. However, sexism and gender awareness were not reported to be frequently imparted gender concepts. Sexism and sexual harassment may result from gender stereotypes. Gender awareness knowledge covers gender stereotypes. The theoretical basis of the term “gender awareness” originates from Marxism and feminism. Gender awareness denotes gender stereotypes and biases [9], as well as the need to recognize, reflect, and criticize power structures and power relations, thereby developing a sense of self-identity. Subsequently, through direct action, changes in the gender power structure can be promoted to achieve gender equity [36,41].

Andersson et al. [9] analyzed the implications of gender awareness and discovered that they constitute more than mere attitudes or beliefs; gender awareness is composed of multiple fundamental and critical gender-related concepts, including gender stereotypes. Course designers and educators for professional health education may combine gender-related concepts more consciously to incorporate into curricula and instruction conceptual categories or themes related to gender. A gender concept category comprises multiple crucial gender-related concepts that require prioritization in learning, and several categories of gender concepts constitute a gender concept theme. According to curriculum planning and instructional design (including class schedules and course duration), teachers may incorporate into their instruction conceptual categories or themes related to gender. This facilitates focus on the learning of core gender concepts as well as creating space for the development of other gender-related concepts.

With an improved conceptualization of gender knowledge (consisting of gender-related concept combinations), course designers or educators can lead students to explore the gender inequality on campus and in clinical practice in depth. Moreover, they can attend to the different needs of individual courses and students’ gender learning and thereby enhance teaching and learning outcomes.

### 4.4. Research Limitations and Future Research Recommendations

The two limitations to this study are as follows. (1) Constraints in time and research funding prevented further qualitative data collection: In this study, self-reported disparities between gender concepts frequently taught and those deemed of top priority for students were identified. Due to time and research funding constraints, this study failed to gain an in-depth understanding of the qualitative answers provided by the participants. Had interviews or in-depth interviews been conducted, the participants may have been able to explain any contradiction. Further research on this is merited. (2) Due to the established sampling criteria, the number of teachers was twice of the number of healthcare professionals: Initially, this study intended to explore potential discrepancies between school education and medical practice and whether undergraduates are sufficiently prepared to apply their gender education in the medical field. However, because the requirements of the sampling criteria were more biased toward academic specialties, the majority of the participants were university professors. Had different sampling criteria been established for teachers and healthcare professionals, more healthcare professionals might have been recruited as participants, more diversified data collected, and the research horizons broadened. For future research on this topic, recruiting patients of different gender identities as the research participants may also be considered. Because of unique patient medical and healthcare experiences, such research may be able to provide different perspectives and insights into gender in professional health education programs.

## 5. Conclusions

Education is the first step to promoting equal health rights for people of all genders. Gender competency is the foundation for eliminating health disparity. In the incorporation of gender into medical and healthcare courses, gender education should be regarded as a professional discipline based on theory and practice rather than as a mere perspective or issue of discussion.

This study showed that the gender concepts of sexism, gender awareness, sexual harassment, the three gender equity acts, and patriarchy were deemed most crucial or top priorities in medical education by 50 medical or nursing teachers and healthcare professionals. Specifically, sexism, gender awareness, and sexual harassment were considered to be core gender knowledge for medical and nursing students to study. The majority of the respondents prioritized education on sexism, gender awareness, sexual harassment, and patriarchy. This study also demonstrated that female participants, having personally experienced real-world gender phenomena in academic and medical settings, were particularly concerned regarding students’ education on gender. In addition, all 50 experts attached value to the teaching of concepts related to patriarchy, the cultivation of students’ structural competency which pinpoint future directions for gender and medical education. Nonetheless, this study discovered consensus, contradictions, and differences in participants’ opinions regarding the gender concepts and knowledge that should be taught when incorporating gender into the medical care curriculum.

Accordingly, this study proposes three practical suggestions. First, according to the gender concepts deemed crucial for medical and nursing students, educators can develop a gender education learning map for medical school students. Course designers or teachers can determine the core gender knowledge required by students and how the learning of other gender-related concepts can be integrated. Second, in response to the diversity in students’ gender knowledge, this study suggests the combination of gender concepts and the use of various categories or themes relating to gender concepts for curriculum design and teaching of gender topics. Designing courses, adopting teaching methods, and planning teaching activities based on combinations of gender concepts enable students with varying levels of gender knowledge to participate in learning activities more effectively.

Third, in addition to the curriculum reform requiring that gender be incorporated into courses, medical universities must also pay attention to the professional development of teachers in gender education. This study emphasizes that, to achieve alignment with the concepts in competency based medical education, the integration into education of fundamental gender concepts should focus on core concepts and the imparting of knowledge in relation to gender, providing an instructional scaffold for subsequent or advanced gender topic learning. Such emphasis also contributes to the integration of professional disciplines of gender and healthcare and learning of competency-based integration ability among students. Therefore, professional development for teachers should enhance their knowledge of feminist theory and gender sociology and their ability to apply gender concepts in curriculum planning and instructional design, thereby enhancing the effectiveness of courses integrating gender with healthcare.

Overall, an understanding of what aspects of gender to teach in medical and healthcare education enables clarification of gender education goals and content, as well as providing a direction for the professional development of teachers. More notably, these findings may serve as a guide for teachers who intend to integrate gender into the curriculum but are subject to time constraints, helping them to focus on key gender concepts and areas of study. This provides a foundation or a stepping stone for students’ gender education, and students are thereby sufficiently prepared for gender competency practice in their future medical professional areas.

## Figures and Tables

**Table 1:** Top 10 gender concepts for medical and nursing students to learn and understand.

Gender Concept	All (*N* = 50)	Sex		Academia/Practice	
		Male (*N* = 18)	Female (*N* = 32)	Teacher (*N* = 35)	Practitioner (*N* = 15)
Sexism	82%	83%	81%	80%	87%
Sexual Harassment	80%	78%	81%	77%	87%
Gender Awareness	76%	73%	78%	80%	67%
Patriarchy	74%	72%	75%	66%	93%
Taiwan’s Three Acts on Gender Equity	72%	72%	72%	71%	73%
LGBTQI	70%	67%	72%	74%	60%
Sexual & Gender-Based Bullying	68%	72%	66%	69%	67%
Sexual & Gender Identity	62%	67%	59%	60%	67%
Autonomy	60%	67%	53%	54%	73%
Objectification	54%	----	63%	57%	----
Oppression & Gender Oppression	54%	61%	50%	----	67%
Body Politics	----	----	54%	54%	----

**Table 2:** Gender concepts prioritized for students’ learning and knowledge.

Rank	Variable	Gender Concept (Weighted Score)
1	All (*N* = 50)	Sexism (460); Gender Awareness (445); Sexual Harassment (435); Taiwan’s Three Acts on Gender Equity (415); Patriarchy (410)
	Male (*N* = 18)	Sexism (165); Taiwan’s Three Acts on Gender Equity (160); Sexual Harassment, Gender Awareness (155); LGBTQI (130)
	Female (*N* = 32)	Sexism (295); Patriarchy, Gender Awareness (290); Sexual Harassment (280); Taiwan’s Three Acts on Gender Equity (255)
	Teacher (*N* = 35)	Gender Awareness (330); Sexism (310); Sexual Harassment (305); Taiwan’s Three Acts on Gender Equity (290); Patriarchy (270)
	Practitioner (*N* = 15)	Sexism (150); Patriarchy (140); Sexual Harassment (130); Taiwan’s Three Acts on Gender Equity (125); Gender Awareness (115)
2	All (*N* = 50)	LGBTQI (365); Sexual & Gender-based Bullying (320); Sexual & Gender Identity (300); Others (290); Autonomy (285)
	Male (*N* = 18)	Sexual & Gender Identity (125); Patriarchy (120); Autonomy, Sexual & Gender-based Bullying (115); Others (105)
	Female (*N* = 32)	LGBTQI (235); Sexual & Gender-based Bullying (205); Objectification (195); Others (185); Male-Identification, Body Politics, Sexual & Gender Identity (175)
	Teacher (*N* = 35)	LGBTQI (265); Others (230); Sexual & Gender-based Bullying (225); Sexual & Gender Identity (210); Autonomy (200)
	Practitioner (*N* = 15)	Abuse of Power (100); LGBTQI (100); Sexual & Gender-based Bullying (95); Heterosexual Hegemony, Heteronormativity, Homophobia, Oppression & Gender Oppression, Sexual & Gender Identity (90)
3	All (*N* = 50)	Objectification, Domestic Violence (245); Oppression & Gender Oppression (235); Heterosexual Hegemony (225); Homophobia (215); Body Politics (210)
	Male (*N* = 18)	Homophobia (95); Oppression & Gender Oppression, Domestic Violence (90); Heteronormativity, Abuse of Power, Heterosexual Hegemony, Patriarchal Dividend (60)
	Female (*N* = 32)	Autonomy (170); Heterosexual Hegemony (165); Domestic Violence (155); Oppression & Gender Oppression; Emotional Labor (145)
	Teacher (*N* = 35)	Objectification (190); Body Politics (170); Domestic Violence (160); Oppression & Gender Oppression (145); Heterosexual Hegemony, Emotional Labor (135)
	Practitioner (*N* = 15)	Autonomy, Domestic Violence (85); Patriarchal Dividend (65); Male-Dominance, Misogyny, Others (60)

1 = Most important (top priority), 2 = Less important (secondary priority), and 3 = Least important (low priority).
